# Redox Status and Aging Link in Neurodegenerative Diseases 2015

**DOI:** 10.1155/2015/494316

**Published:** 2015-08-02

**Authors:** Verónica Pérez de la Cruz, Sathyasaikumar V. Korrapati, José Pedraza Chaverrí

**Affiliations:** ^1^Departamento de Neuroquímica, Instituto Nacional de Neurología y Neurocirugía Manuel Velasco Suárez, S.S.A., 14269 México, DF, Mexico; ^2^Maryland Psychiatric Research Center, University of Maryland School of Medicine, Baltimore, MD 21228, USA; ^3^Departamento de Biología, Facultad de Química, Universidad Nacional Autónoma de México, 04510 México, DF, Mexico

Aging is a multifactorial degenerative process and is characterized by progressive deterioration in physiological functions and metabolic processes, changes that drive numerous age-related disorders. Within cellular alterations are found oxidative stress, inflammation, and mitochondrial dysfunction, factors that converge in the aging and conduce to cognitive decline and other pathologies. Specially, the brain is susceptible to alterations in the redox environment due its own properties (high concentrations of polyunsaturated fatty acids, high oxygen demand, and its poor antioxidant system compared with other organs). During the aging, the activity of antioxidant enzymes decreases and oxidative markers are elevated in various organs, particularly in the brain. This special issue contributes to understanding of the mechanism involved during the aging process and provides the recent findings in this paradigm.

The original research article by N. Izuo and coworkers shows us that superoxide dismutase-2 (SOD2) knockout mice have an increase in oxidative markers and alteration in the mitochondrial complex II that was associated with the development of neurodegeneration. These findings strongly suggest that SOD2 plays an important role in cellular defense against oxidative damage and could be a potential target in the aging considering that SOD2 is a mitochondrial antioxidant enzyme.

The review by M. Luca et al. is focused on the role of oxidative stress in Alzheimer's disease and vascular dementia. The authors also show the recent evidence regarding the use of antioxidant therapy and discuss new therapeutic strategies, such as the potential of inhibitors of heat shock proteins in the prevention and treatment of these pathologies.

Too many factors are involved during the aging; one of these important factors involves the mitochondrial function, which is a key for the energetic metabolism and its impairment could drive to oxidative stress and cell death. A. Schloesser and coworkers showed here that the diet with tocotrienols complexed with *γ*-cyclodextrin improves the mitochondrial membrane potential and ATP concentrations in the brains of aged mice. With these findings, many possibilities are open to study, which may lead to evaluating the mechanisms by which this diet improves mitochondrial parameters evaluated.

An interesting review by L. Szalárdy and coworkers also enhances the importance of mitochondrial function and describes step by step how mitochondria participate in the ROS production and the vicious circle with oxidative phosphorylation when mitochondria are injured. The authors also describe the potential therapeutic relevance of PGC-1*α* in neurological disorders.

Finally, the original research article by M. Rubio-Osornio and coworkers shows the effect of epicatechin on the oxidative and behavioral damage induced by MPP+, considering that this polyphenol possesses scavenging properties. Their data show a neuroprotective effect of epicatechin in this neurotoxic model and suggest that the protective effect may be due, at least in part, to the induction of an increase of Cu,Zn-SOD activity.

All the items of this special issue have included an emphasis on mitochondrial function, oxidative stress during the aging, and related diseases. We need to maintain in mind that all these factors are interconnected and cannot be easily separated from each other; for this reason the manuscripts in this special issue are relevant to clarify and understand them.

*Verónica Pérez de la Cruz* *Verónica Pérez de la Cruz* *Sathyasaikumar V. Korrapati* *Sathyasaikumar V. Korrapati* *José Pedraza Chaverrí* *José Pedraza Chaverrí*

